# Evidence of integrated primary-secondary health care in low-and middle-income countries: protocol for a scoping review

**DOI:** 10.1186/s13643-020-01514-3

**Published:** 2020-11-09

**Authors:** Md Zabir Hasan, Shalini Singh, Dinesh Arora, Nishant Jain, Shivam Gupta

**Affiliations:** 1 Department of International Health, Johns Hopkins Bloomberg School of Public Health, Baltimore, MD USA; 2 Deutsche Gesellschaft für Internationale Zusammenarbeit (GIZ), New Delhi, India

**Keywords:** Integrated care, Integrated primary-secondary care, Health systems, Ayushman Bharat, Scoping review, Low- and middle-income countries, India

## Abstract

**Background:**

Integrated care is a people-centered health delivery approach that ensures the comprehensiveness, quality, and continuity of service across the settings and levels of health systems. The World Health Organization (WHO) recommends integration across levels and building-blocks of health systems as a prerequisite of Universal Health Coverage (UHC). While health systems of low- and middle-income countries (LMICs) are often fragmented and led by siloed service delivery structure, several LMICs—including India—have attempted health system integration. Several systematic reviews of evidence on healthcare integration from developed countries exist, but no synthesis from LMICs was reported to date. This review will overview the existing evidence of primary-secondary care integration (PSI) in the context of LMICs, aiming to support policy decisions for the effective integration of health delivery systems in India.

**Methods:**

The review will be conducted following the six steps recommend by Arksey and O'Malley. Scientific and grey literature will be systematically selected from PubMed, Embase, Scopus, Web of Science, Global Index Medicus, and electronic repositories (such as WHO, World Bank, Health Policy Plus, and OpenGrey). Using a comprehensive search strategy, literature written in English and published between 2000 and 2020 will be selected, and two independent authors will screen their titles and abstracts. The result will be charted using a data extraction form and reported using tables, figures, and narrative forms.

**Discussion:**

No ethical approval is necessary for the review. The final report will be developed with the consultation of other stakeholders and disseminated through workshops, conference papers, and peer review articles. The review will serve as a guiding tool to approach, implement, and test the PSI models in India and other LMICs.

**Scoping review registration:**

https://osf.io/kjhzt.

**Supplementary Information:**

The online version contains supplementary material available at 10.1186/s13643-020-01514-3.

## Background

### Integrated primary-secondary care system

Health service integration is seen by the World Health Organization (WHO) as an essential requirement to achieve Universal Health Coverage (UHC) [1]. According to WHO, an integrated health service delivery is defined as:

… an approach to strengthen people-centered health systems through the promotion of the comprehensive delivery of quality services across the life-course, designed according to the multidimensional needs of the population and the individual and delivered by a coordinated multidisciplinary team of providers working across settings and levels of care… … with feedback loops to continuously improve performance and to tackle upstream causes of ill health and to promote well-being through intersectoral and multisectoral actions [2].

**Fig. 1:**
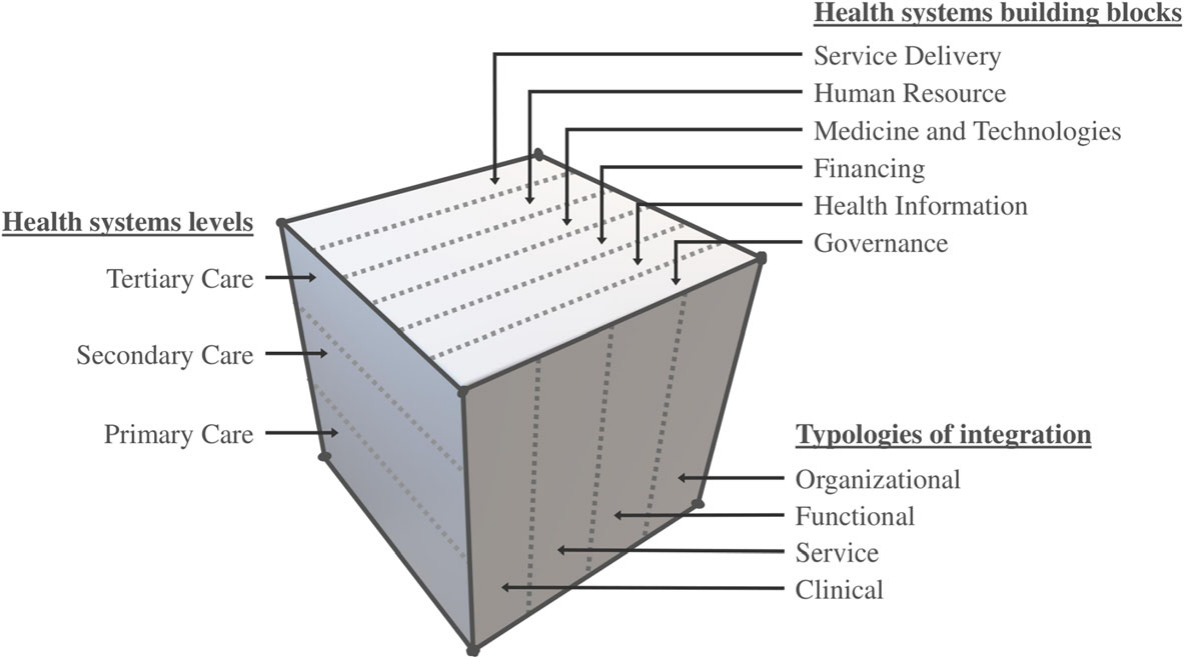
Complexity of vertical integration within health systems

The definition itself reveals the multidimensionality of the integrated care system. As Fig. 1 indicates, integration can happen across the level of care (such as primary, secondary, or tertiary), between the building blocks of the health systems (service delivery, human resource, medicine and technologies, financing, health information, or governance) and even across geography or time-span [3]. Moreover, based on its nature, integrated care can be divided into (a) Organizational: different health service organizations are merged for care coordination; (b) Functional: non-clinical services are related with integration (such as administration, electrical supply, etc.); (c) Service: different type or genre of service merged at facility level using a multi-disciplinary team; and lastly (d) Clinical: application of shared guideline and protocol to treat comorbidity or ensuring continuum of care [4, 5].

The primary-secondary care integration (PSI) is a part of vertical integration where the primary healthcare system is efficiently connected with the rest of the healthcare delivery structure, but most importantly, with secondary care. Within the hierarchical structure of the health system, PSI can bring “closer-to-community services” within broader management (organizational integration) or provide the opportunity for functional, clinical, or service level integration [4]. It opens the prospect for organized service delivery between primary care provider and specialist, leading to early detection of disease, improved quality of care, better follow-up, and patient outcome [6–10]—however—effectiveness of the integration can vary by context and interventions [11].

### Indian health systems in transition

While high-income countries are already moving to integrated service configurations and delivery structure, low- and middle-income countries (LMICs) have yet to significantly implement integrated care in their public health systems, mostly because of the complexities of implementation in a context where healthcare infrastructure and human resources for health are inadequate [1]. For example, the Government of India launched the Ayushman Bharat (AB) program in 2018 to restructure the public financing for healthcare and service delivery mechanisms toward UHC [12].

The AB program consists of two unique—but complementary—components: (a) the Pradhan Mantri Jan Arogya Yojana (PM-JAY) and (b) scaling up Health and Wellness Centres (HWCs) [12]. PM-JAY is considered the largest health assurance program globally, providing financial protection for 500 million of the poorest and vulnerable individuals [13]. Under this scheme, a family will be covered up to 500,000 Rupee (approximately 6978 USD, considering the exchange rate of 71.65 Rupee = 1 USD on 2 January 2020) per year. This will cover approximately 1393 procedures, including doctor’s fees, drugs, supplies, diagnostics, room charges, etc. However, these benefits are eligible for only secondary and tertiary care services [14].

While implementing PM-JAY, the national and state health systems of India are transitioning through several structural integrations. PM-JAY is administered by a separate National Health Authority (NHA) at the national level. However, the individual state retains the flexibility to implement the scheme through the State Health Agency (SHA). State governments must contribute 40% of the cost of running the program and are encouraged to merge state-funded insurance schemes with PM-JAY to expand the risk pool. All public secondary and tertiary hospitals are automatically included under PM-JAY. The private health sector has been integrated within PM-JAY by empaneling private hospitals based on defined criteria linked to specific service packages [15]. Further, functional integrations are also evident within the PM-JAY scheme through the portability of services across states.

While implementing the AB program, the Government of India is establishing 150,000 HWCs to prepare the primary healthcare delivery system for the emerging epidemiologic transition [16]. HWCs are located within 30 min, traveling distance from any community. HWCs will provide an expanded range of services that will be available, including reproductive, maternal, and child health, preventive and curative care for communicable and chronic diseases, and support for mental health, etc. [16]. To make it as the first point of contact closer to the community, existing rural clinics—designated as sub-centers—and rural and urban primary health centers (PHCs) will be upgraded as HWCs to deliver comprehensive primary health care (CPHC) [17].

Since its launch till February 2020, PM-JAY has empaneled over 21 thousand hospitals, issued more than 12 million beneficiary cards, and provided financial support for 3.6 million beneficiaries [13, 14]. Simultaneously, over 30 thousand HWCs have been established by transforming 13,516 Sub-centers, 13,417 PHCs, and 3076 urban PHCs [16]. However, the critical link between primary-secondary care provision is still missing, as PM-JAY does not yet provide financial support at these primary level facilities. Moreover, the care continuum pathway is still evolving beyond HWCs, owing to its early implementation stage.

### Rationale of the review

The AB program’s progress has been promising so far, yet some critical design challenges have been identified. The fact that PM-JAY will not cover primary care, it is even more imperative to plan strategies under AB to increase the accountability of HWCs to deliver CPHC so that people do not bypass preventive and promotive services offered by HWCs. This will limit efforts of secondary prevention and lead to increased care-seeking from secondary or tertiary care facilities. As the demand for service will increase, the overburdened secondary or tertiary care facilities will face issues with the quality of care. More importantly, increasing demand will jeopardize the fiscal sustainability of PM-JAY [18].

Thus, the role of HWCs is paramount in this case. As the first point of contact, HWCs are mandated to keep the population healthy through prevention strategies and extensive lifestyle counseling. This will contribute to a lower frequency of procedures or curative care events. Further strengthening of HWCs by focusing on the continuum of care—starting from early initiation of preventive care, judicious use of curative care, proper referral, follow up care, and educating the beneficiaries about PM-JAY—need to be prioritized [19]. This calls for effective integration of primary and secondary care between HWCs and other secondary-level public and private providers within the AB program.

Therefore, to enable implementation of PSI models within the AB system, there is a need to identify design features that can be adopted within the healthcare delivery systems as part of AB to enable a sound linkage across different service provision levels. Other existing structural deficiencies of the Indian health care delivery system—such as governance, stewardship, quality of care, and health system organization—will also impose additional layers of complexity. Thus, looking at the global best practices and experience of health service integration is the most pragmatic way forward.

Several systemic and scoping reviews were already conducted exploring the PSI models; however, the majority of those studies were conducted in the context of developed countries [7, 8, 20–22]. Some evidence from LMICs has already shown that vertical integration of primary and secondary care has a strong potential to increase access, reduce cost, and improve quality and health outcomes [21, 22]. There is an insufficient exploration of critical aspects of design, implementation of strategies, assessment of PSI models, and consolidation of evidence from other LMICs. A scoping review of available evidence from LMICs will help to understand the design elements and integration processes that have been used to thrive in an integrated care model for UHC. As no prior synthesis has been undertaken on this topic—exclusively—in the context of LMIC, scoping review is an appropriate method [23]. The methodological plasticity of scoping review will allow us to evaluate a breadth of contents—including qualitative, quantitative, mixed-method studies, reports, and other grey literature—to map, organize, identify, and report the current knowledge base from LMIC countries. This exercise will draw the attention of stakeholders and support in building momentum toward a systemic reform for PSI for health service delivery in India [24].

## Objectives

The following three questions will guide the inquiry, analysis, and consolidation of evidence in our scoping review:

1. How are the PSI models defined during implementation in the context of LMICs?
2. What are the characteristic features of organizational and operational components of PSI models in LMIC settings?
3. What is the current evidence regarding the influence of PSI models on the health systems and population health in LMIC settings?

## Methods

To conduct the scoping review, we will consider the six stages recommended by Arksey and O'Malley, and Levac et al. [25, 26]. This protocol has been registered within the Open Science Framework (Registration link: https://osf.io/kjhzt). Besides, it is developed by following the guidance of Preferred Reporting Items for Systematic Reviews and Meta-Analyses Protocols (PRISMA-P) [27] (the see checklist is provided as Additional File 1). The result will be reported in accordance with the reporting guidance provided in the Preferred Reporting Items for Systematic Reviews and Meta-analyses extension for Scoping Reviews (PRISMA-ScR) statement [28].

### Stage 1: identifying the research questions

Collaborating with researchers from Johns Hopkins Bloomberg School of Public Health (JHSPH) and Deutsche Gesellschaft für Internationale Zusammenarbeit (GIZ), India, three research questions are proposed with a broader scope while having a precise aim, listed in the previous section.

### Stage 2: identifying relevant literature

The literature search will include a broad range of terms and keywords related to three concepts—“integrated care model,” “primary and secondary care system,” and “low- and middle-income countries.” The primary source of evidence will be a structured literature search of five electronic databases (from 2000 to 2020): PubMed/MEDLINE, EMBASE, Scopus, Web of Science, and Global Index Medicus. The secondary source of potentially relevant material will be a search of the grey literature, including institutional websites (e.g., WHO and World Bank), Health Policy Plus, Google Scholar, and OpenGrey. Besides, hand-searching of the reference lists will be performed for selected articles and policy reports. If necessary, subject matter experts and prolific authors in the field will be contacted based on the study’s feasibility and necessity.

The review team will lead the design and implementation of the search strategy, and we will actively consult with a health information specialist [29]. A draft search strategy for PubMed/MEDLINE is provided in Additional File 2. After implementing the search, the title and abstracts will be downloaded, and citations will be imported into Covidence systematic review software (covidence.org). At this stage, we will remove the duplicates and organize the search records to review their titles and abstracts.

### Stage 3: screening and selection procedure

We have used the “Population-Concept-Context (PCC)” framework [30] to align our study selection process with the specific research questions mentioned in the previous section. The preliminary inclusion and exclusion criteria for the screening are presented in Table 1.

**Table 1:** Inclusion and exclusion criteria for the record selection process

	Inclusion criteria	Exclusion criteria
Concept	• Integrated primary-secondary health care model	• Article lacking evidence or discussion on the primary-secondary care integration (Example: A study may report the prevalence of obesity-related comorbidity and then recommending an integrated primary-secondary health care model as a recommendation. This will be excluded during the selection process)
Context and population	• 31 low-income countries and 47 lower-middle-income countries based on the World Bank's classification^a^	
Document type	• Peer-reviewed journal articles • Grey literature (such as policy brief or programmatic reports) • Quantitative, qualitative, mixed or multimethod research, policy analysis, methodology paper	• Chart reviews, opinion papers, case reports, editorials • Conference proceedings and posters
Time frame	• 2000 - 2020	
Reporting characteristics	• Articles are written in English • Complete articles that have been published	• Article not published in English

https://datahelpdesk.worldbank.org/knowledgebase/articles/906519-world-bank-country-and-lending-groups

^a^Countries Gross domestic product (GDP) less than $3995

The review will include a wide range of documents, such as peer-reviewed publications, methodological papers, and grey literature, published in the English language from 2000 to 2020. However, we decided not to include chart reviews, opinion papers, case reports, editorials, and conference proceedings. Most importantly, to be included in the review, the document or article will need to include evidence on the primary-secondary integrated health care model in low- and middle-income countries, which we have considered as (see supplemental material Additional File 2 for further details):

The approach toward vertical integration of primary and secondary healthcare systems, which requires a set of coordinated strategies that involve streamlining the organizational arrangements, functional processes, service delivery apparatuses, clinical operations, and community-health facility interfaces—either by implementing independently or in any specific combinations—for incorporating secondary care functions within the primary care settings or vice versa, enabling upstream and/or downstream restructuring by augmenting health systems resources—within one setting or across health facilities—to provide evidence-based, people-centered and high-quality healthcare service and, simultaneously, to improve the performance of health systems.

Following these criteria, two independent reviewers will screen the title and the abstract (or executive summary) of the searched documents. For those eligible peer-reviewed journal articles and grey literature, which will appear to represent the subject of our scoping review, copies of the full documents will be obtained. Next, the reviewers will read the full document and decide to include the article/report in the review process. The research team will regularly review the result of the screening process and discuss the discrepancies. In the case of an undisputed disagreement, a senior researcher will take the role of arbitrator to resolve the issue. We acknowledge that literature search and screening is an iterative process, and we will pragmatically adapt the search criteria if necessary and reiterate the screening process [25].

### Stage 4: charting the data

Full-text articles included in the scoping review will be re-appraised, and information will be charted using a data extraction form [30]. Focusing on the research question identified in stage one, we have developed a data extraction form to summarize the evidence from the document. The themes and variables included in the data extraction form are presented in Table 2, and the extended version of the data extraction form is provided in Additional File 2 as supplemental material.

**Table 2:** Summary of the data extraction form to chart the evidence

Data extraction themes	Extracted data
Study characteristics	Source
	Title
	Authors
	Year
	Country name
	Country type (World Bank’s classification^a^)
	WHO region
	Study populations
	Study location
	Study design and methodology
Scoping review specific	Definition of integrated care
	Typologies of integration
	Type of service integrated (if applicable)
	Health systems building blocks integrated (if applicable)
	Mechanism of integration (if applicable)
	Structure of integration (if applicable)
	Intensity of integration (if applicable)
	Organizational and operational components
	Success
	Facilitators
	Bottlenecks
	Unintended consequences

https://datahelpdesk.worldbank.org/knowledgebase/articles/906519-world-bank-country-and-lending-groups

^a^Countries Gross domestic product (GDP) less than $3995

Two distinct sets of information will be charted using the data extraction form. The first set of information is related to the characteristics of the article, which will include the source of the article, title, authors, publication year, country where the study was conducted, or the evidence was generated, country type (low-income or lower-middle-income), WHO region, study populations, study location (urban or rural), design and methodology of the study, etc.

The second set of information is specific to this scoping review which are, but not limited to, definition of integrated care, typologies of integration, type of service integrated, health systems building blocks integrated, mechanism of integration, the structure of integration, the intensity of integration, organizational and operational components of integration, success, facilitators, bottlenecks, and unintended consequences. Detailed descriptions of each of the scoping review specific thematic areas are presented in the supplementary materials of Additional File 2.

A team of two researchers will conduct the data charting process. The process will start with a pilot exercise, where the two data extractors will independently chart the data from the same five eligible articles in parallel using an initial draft of data extraction from (developed based on Table 2). A workshop will be conducted in the presence of a third senior researcher as an arbitrator to triangulate the extracted data to streamline and harmonize the data charting process between the two researchers. Next, the eligible articles will be divided among the two researchers for completing the charting process. After completing every ten articles, the senior researcher will moderate a review meeting to go over the extraction process and resolve any impending or unintended issues in the charting process. During this stage, if additional details regarding an article or report are required, we will try to reach out to the investigators for additional information while pragmatically considering the time and resources required for this process.

### Stage 5: collating and reporting the results

In the fifth stage, the charted information will be summarized into thematic areas and reported in a narrative format with tables and illustrations. We will present an overview of the material included in the screening and the review process. Based on the number of articles, we will use Dedoose (dedoose.com) to extract the emergent themes and patterns from the data if necessary. The result will be clustered and presented to explore the geographic, socioeconomic, and health system variation across the countries.

### Stage 6: expert consultation

While Arksey and O'Malley suggested expert consultation as optional for scoping review [25], we agree with Levac et al. [26] that this stage is essential for finalizing the scoping review. We also intend to perform a consultation exercise after developing the initial report of the scoping review. This scoping review is commissioned to inform and provide pragmatic recommendations to the AB program to lead a discussion for instilling PSI models as an instrument for achieving UHC. Thus, we need to organize the result and collate the evidence of our review to be palatable for the policymakers and public health practitioners in India. We will develop a roster of researchers from JHSPH, GIZ, and other organizational networks, practitioners, and public health policymakers for this consultation process to strengthen the review [31]. This is a critical component of our study as this part links the evidence with the ground reality of the Indian health system. Translating the finding of the review into pragmatic recommendations for adopting a PSI model is critical for this formative exercise.

## Discussion

Ethical approval will not be necessary for the scoping review. The required information will be obtained from the publicly available literature on integrated health care models that have been adopted in LMICs, and no primary data will be collected. The investigators will also maintain a research log to record all necessary changes and methodological decisions taken while conducting the scoping review. Furthermore, the details of any changes made to the protocol of this scoping review will be outlined in the Open Science Framework, in addition to reporting in the final manuscript.

Before embarking on the journey to conduct this review, we have anticipated two operational challenges. Firstly, the three core concepts liked to the objective of this scoping review (“integrated care model,” “primary and secondary care system,” and “low- and middle-income countries”) are relatively broad and somehow ambiguous. We anticipated that the implementation of the search strategy would produce a considerable breadth of documents, which has possible implications on the study timeframe. Secondly, as a part of the inclusion criteria, we will include studies with a wide range of methodological variation. Though this variability will not affect the title and abstract review or full-text screening process, synthesis and collation of the wide range of evidence in a harmonized way will be a challenge.

We also want to acknowledge a few anticipated limitations of this review. While structuring this scoping review, we have decided to include the five major literature databases that encompass most health science-related articles. However, there is always a possibility of missing some relevant documents related to PSI models [32]. We will actively try to mitigate this limitation by exploring additional databases of the grey literature. However, unlike a systematic review, developing an all-encompassing search strategy and collating all relevant studies is impossible for scoping reviews [33]. Besides, excluding the documents that are not published in English will also lead to some study attrition. Lastly, while not a limitation, it is essential to emphasize that we will not assess the quality of the evidence during the review process or compare the robustness or generalizability of different PSI models [24, 25, 30].

This scoping review forms a part of the overarching study to-explore the scope of PSI models under the AB Program in India. Thus, we are particularly interested in building an efficient dissemination strategy for this scoping review. The dissemination process will start with sharing the initial findings of this review with other researchers, practitioners, and public health policymakers within and beyond our organizational networks as part of the consultation exercise to enhance the review’s quality. The final report will be disseminated through a workshop involving critical national and state-level stakeholders for the AB program in India. The findings will serve as an advocacy tool for rolling out PSI for UHC in India. We will also develop publication as peer-reviewed journal articles and share the result via dissemination events such as conferences and online fora.

An integrated primary, secondary, and tertiary health care model is an essential strategy to transition toward UHC. This scoping review will inform policy decisions, legislative and financing frameworks, changes in the health service organization, use of information systems, and health teams’ orientation that will be needed to facilitate PSI in India. This study will serve as a foundation for modeling and implementing the efforts of PSI under the AB program.

## Supplementary Information

**Additional file 1.**

**Additional file 2.**

## Data Availability

Not applicable.

## Abbreviations

WHO: World Health Organization
UHC: Universal Health Coverage
LMICs: Low- and middle-income countries
PSI: Primary-secondary care integration
AB: Ayushman Bharat
PM-JAY: Pradhan Mantri Jan Arogya Yojana
HWCs: Health and Wellness Centres
USD: United States Dollar
NHA: National Health Authority
SHA: State Health Agency
PHCs: Primary health centers
CPHC: Comprehensive primary health care
PRISMA-ScR: Preferred Reporting Items for Systematic Reviews and Meta-Analyses’ Extension for Scoping Reviews
JHSPH: Johns Hopkins Bloomberg School of Public Health
GIZ: Deutsche Gesellschaft für Internationale Zusammenarbeit
PRISMA-P: Preferred Reporting Items for Systematic Reviews and Meta-Analyses Protocols
