# Supplementary material for: Evidence of integrated primary-secondary health care in low-and middle-income countries: protocol for a scoping review
Source: Syst Rev. 2020 Nov 9;9:260. doi: 10.1186/s13643-020-01514-3 (PMC7654598; doi:10.1186/s13643-020-01514-3)
Supplement: Supplementary file 2 — Additional file 2. [file 13643_2020_1514_MOESM2_ESM.docx]

***Evidence of integrated primary-secondary health care in
low-and middle-income countries: protocol for a scoping review***

***Supplemental Materials***

**Supplemental Material 1:** Low- and middle-income economies Pubmed filter (World Bank - 2013)

"developing country"[tiab] OR "developing countries"[tiab] OR "developing nation"[tiab] OR "developing nations"[tiab] OR "developing population"[tiab] OR "developing populations"[tiab] OR "developing world"[tiab] OR "less developed country"[tiab] OR "less developed countries"[tiab] OR "less developed nation"[tiab] OR "less developed nations"[tiab] OR "less developed population"[tiab] OR "less developed populations"[tiab] OR "less developed world"[tiab] OR "lesser developed country"[tiab] OR "lesser developed countries"[tiab] OR "lesser developed nation"[tiab] OR "lesser developed nations"[tiab] OR "lesser developed population"[tiab] OR "lesser developed populations"[tiab] OR "lesser developed world"[tiab] OR "under developed country"[tiab] OR "under developed countries"[tiab] OR "under developed nation"[tiab] OR "under developed nations"[tiab] OR "under developed population"[tiab] OR "under developed populations"[tiab] OR "under developed world"[tiab] OR "underdeveloped country"[tiab] OR "underdeveloped countries"[tiab] OR "underdeveloped nation"[tiab] OR "underdeveloped nations"[tiab] OR "underdeveloped population"[tiab] OR "underdeveloped populations"[tiab] OR "underdeveloped world"[tiab] OR "middle income country"[tiab] OR "middle income countries"[tiab] OR "middle income nation"[tiab] OR "middle income nations"[tiab] OR "middle income population"[tiab] OR "middle income populations"[tiab] OR "low income country"[tiab] OR "low income countries"[tiab] OR "low income nation"[tiab] OR "low income nations"[tiab] OR "low income population"[tiab] OR "low income populations"[tiab] OR "lower income country"[tiab] OR "lower income countries"[tiab] OR "lower income nation"[tiab] OR "lower income nations"[tiab] OR "lower income population"[tiab] OR "lower income populations"[tiab] OR "underserved country"[tiab] OR "underserved countries"[tiab] OR "underserved nation"[tiab] OR "underserved nations"[tiab] OR "underserved population"[tiab] OR "underserved populations"[tiab] OR "underserved world"[tiab] OR "under served country"[tiab] OR "under served countries"[tiab] OR "under served nation"[tiab] OR "under served nations"[tiab] OR "under served population"[tiab] OR "under served populations"[tiab] OR "under served world"[tiab] OR "deprived country"[tiab] OR "deprived countries"[tiab] OR "deprived nation"[tiab] OR "deprived nations"[tiab] OR "deprived population"[tiab] OR "deprived populations"[tiab] OR "deprived world"[tiab] OR "poor country"[tiab] OR "poor countries"[tiab] OR "poor nation"[tiab] OR "poor nations"[tiab] OR "poor population"[tiab] OR "poor populations"[tiab] OR "poor world"[tiab] OR "poorer country"[tiab] OR "poorer countries"[tiab] OR "poorer nation"[tiab] OR "poorer nations"[tiab] OR "poorer population"[tiab] OR "poorer populations"[tiab] OR "poorer world"[tiab] OR "developing economy"[tiab] OR "developing economies"[tiab] OR "less developed economy"[tiab] OR "less developed economies"[tiab] OR "lesser developed economy"[tiab] OR "lesser developed economies"[tiab] OR "under developed economy"[tiab] OR "under developed economies"[tiab] OR "underdeveloped economy"[tiab] OR "underdeveloped economies"[tiab] OR "middle income economy"[tiab] OR "middle income economies"[tiab] OR "low income economy"[tiab] OR "low income economies"[tiab] OR "lower income economy"[tiab] OR "lower income economies"[tiab] OR "low gdp"[tiab] OR "low gnp"[tiab] OR "low gross domestic"[tiab] OR "low gross national"[tiab] OR "lower gdp"[tiab] OR "lower gnp"[tiab] OR "lower gross domestic"[tiab] OR "lower gross national"[tiab] OR lmic[tiab] OR lmics[tiab] OR "third world"[tiab] OR "lami country"[tiab] OR "lami countries"[tiab] OR "transitional country"[tiab] OR "transitional countries"[tiab] OR Africa[tiab] OR Asia[tiab] OR Caribbean[tiab] OR West Indies[tiab] OR South America[tiab] OR Latin America[tiab] OR Central America[tiab] OR "Atlantic Islands"[tiab] OR "Commonwealth of Independent States"[tiab] OR "Pacific Islands"[tiab] OR "Indian Ocean Islands"[tiab] OR "Eastern Europe"[tiab] OR Afghanistan[tiab] OR Albania[tiab] OR Algeria[tiab] OR Angola[tiab] OR Antigua[tiab] OR Barbuda[tiab] OR Argentina[tiab] OR Armenia[tiab] OR Armenian[tiab] OR Aruba[tiab] OR Azerbaijan[tiab] OR Bahrain[tiab] OR Bangladesh[tiab] OR Barbados[tiab] OR Benin[tiab] OR Byelarus[tiab] OR Byelorussian[tiab] OR Belarus[tiab] OR Belorussian[tiab] OR Belorussia[tiab] OR Belize[tiab] OR Bhutan[tiab] OR Bolivia[tiab] OR Bosnia[tiab] OR Herzegovina[tiab] OR Hercegovina[tiab] OR Botswana[tiab] OR Brasil[tiab] OR Brazil[tiab] OR Bulgaria[tiab] OR Burkina Faso[tiab] OR Burkina Fasso[tiab] OR Upper Volta[tiab] OR Burundi[tiab] OR Urundi[tiab] OR Cambodia[tiab] OR Khmer Republic[tiab] OR Kampuchea[tiab] OR Cameroon[tiab] OR Cameroons[tiab] OR Cameron[tiab] OR Camerons[tiab] OR Cameroun[tiab] OR Cape Verde[tiab] OR Central African Republic[tiab] OR Chad[tiab] OR Chile[tiab] OR China[tiab] OR Colombia[tiab] OR Comoros[tiab] OR Comoro Islands[tiab] OR Comores[tiab] OR Mayotte[tiab] OR Congo[tiab] OR Zaire[tiab] OR Costa Rica[tiab] OR Cote d'Ivoire[tiab] OR Ivory Coast[tiab] OR Croatia[tiab] OR Cuba[tiab] OR Cyprus[tiab] OR Czechoslovakia[tiab] OR Czech Republic[tiab] OR Slovakia[tiab] OR Slovak Republic[tiab] OR Djibouti[tiab] OR French Somaliland[tiab] OR Dominica[tiab] OR Dominican Republic[tiab] OR East Timor[tiab] OR East Timur[tiab] OR Timor Leste[tiab] OR Ecuador[tiab] OR Egypt[tiab] OR United Arab Republic[tiab] OR El Salvador[tiab] OR Eritrea[tiab] OR Estonia[tiab] OR Ethiopia[tiab] OR Fiji[tiab] OR Gabon[tiab] OR Gabonese Republic[tiab] OR Gambia[tiab] OR Gaza[tiab] OR Georgia Republic[tiab] OR Georgian Republic[tiab] OR Ghana[tiab] OR Gold Coast[tiab] OR Greece[tiab] OR Grenada[tiab] OR Guatemala[tiab] OR Guinea[tiab] OR Guam[tiab] OR Guiana[tiab] OR Guyana[tiab] OR Haiti[tiab] OR Honduras[tiab] OR Hungary[tiab] OR India[tiab] OR Maldives[tiab] OR Indonesia[tiab] OR Iran[tiab] OR Iraq[tiab] OR Isle of Man[tiab] OR Jamaica[tiab] OR Jordan[tiab] OR Kazakhstan[tiab] OR Kazakh[tiab] OR Kenya[tiab] OR Kiribati[tiab] OR Korea[tiab] OR Kosovo[tiab] OR Kyrgyzstan[tiab] OR Kirghizia[tiab] OR Kyrgyz Republic[tiab] OR Kirghiz[tiab] OR Kirgizstan[tiab] OR "Lao PDR"[tiab] OR Laos[tiab] OR Latvia[tiab] OR Lebanon[tiab] OR Lesotho[tiab] OR Basutoland[tiab] OR Liberia[tiab] OR Libya[tiab] OR Lithuania[tiab] OR Macedonia[tiab] OR Madagascar[tiab] OR Malagasy Republic[tiab] OR Malaysia[tiab] OR Malaya[tiab] OR Malay[tiab] OR Sabah[tiab] OR Sarawak[tiab] OR Malawi[tiab] OR Nyasaland[tiab] OR Mali[tiab] OR Malta[tiab] OR Marshall Islands[tiab] OR Mauritania[tiab] OR Mauritius[tiab] OR Agalega Islands[tiab] OR "Melanesia"[tiab] OR Mexico[tiab] OR Micronesia[tiab] OR Middle East[tiab] OR Moldova[tiab] OR Moldovia[tiab] OR Moldovian[tiab] OR Mongolia[tiab] OR Montenegro[tiab] OR Morocco[tiab] OR Ifni[tiab] OR Mozambique[tiab] OR Myanmar[tiab] OR Myanma[tiab] OR Burma[tiab] OR Namibia[tiab] OR Nepal[tiab] OR Netherlands Antilles[tiab] OR New Caledonia[tiab] OR Nicaragua[tiab] OR Niger[tiab] OR Nigeria[tiab] OR Northern Mariana Islands[tiab] OR Oman[tiab] OR Muscat[tiab] OR Pakistan[tiab] OR Palau[tiab] OR Palestine[tiab] OR Panama[tiab] OR Paraguay[tiab] OR Peru[tiab] OR Philippines[tiab] OR Philipines[tiab] OR Phillipines[tiab] OR Phillippines[tiab] OR Poland[tiab] OR Portugal[tiab] OR Puerto Rico[tiab] OR Romania[tiab] OR Rumania[tiab] OR Roumania[tiab] OR Russia[tiab] OR Russian[tiab] OR Rwanda[tiab] OR Ruanda[tiab] OR Saint Kitts[tiab] OR St Kitts[tiab] OR Nevis[tiab] OR Saint Lucia[tiab] OR St Lucia[tiab] OR Saint Vincent[tiab] OR St Vincent[tiab] OR Grenadines[tiab] OR Samoa[tiab] OR Samoan Islands[tiab] OR Navigator Island[tiab] OR Navigator Islands[tiab] OR Sao Tome[tiab] OR Saudi Arabia[tiab] OR Senegal[tiab] OR Serbia[tiab] OR Montenegro[tiab] OR Seychelles[tiab] OR Sierra Leone[tiab] OR Slovenia[tiab] OR Sri Lanka[tiab] OR Ceylon[tiab] OR Solomon Islands[tiab] OR Somalia[tiab] OR Sudan[tiab] OR Suriname[tiab] OR Surinam[tiab] OR Swaziland[tiab] OR Syria[tiab] OR Syrian[tiab] OR Tajikistan[tiab] OR Tadzhikistan[tiab] OR Tadjikistan[tiab] OR Tadzhik[tiab] OR Tanzania[tiab] OR Thailand[tiab] OR Togo[tiab] OR Togolese Republic[tiab] OR Tonga[tiab] OR Trinidad[tiab] OR Tobago[tiab] OR Tunisia[tiab] OR Turkey[tiab] OR Turkmenistan[tiab] OR Turkmen[tiab] OR Tuvalu[tiab] OR Uganda[tiab] OR Ukraine[tiab] OR Uruguay[tiab] OR USSR[tiab] OR Soviet Union[tiab] OR Union of Soviet Socialist Republics[tiab] OR Uzbekistan[tiab] OR Uzbek OR Vanuatu[tiab] OR New Hebrides[tiab] OR Venezuela[tiab] OR Vietnam[tiab] OR Viet Nam[tiab] OR West Bank[tiab] OR Yemen[tiab] OR Yugoslavia[tiab] OR Zambia[tiab] OR Zimbabwe[tiab] OR Rhodesia[tiab] OR Developing Countries[Mesh] OR Africa[Mesh:noexp] OR Africa, Northern[Mesh:noexp] OR Africa South of the Sahara[Mesh:noexp] OR Africa, Central[Mesh:noexp] OR Africa, Eastern[Mesh:noexp] OR Africa, Southern[Mesh:noexp] OR Africa, Western[Mesh:noexp] OR Asia[Mesh:noexp] OR Asia, Central[Mesh:noexp] OR Asia, Southeastern[Mesh:noexp] OR Asia, Western[Mesh:noexp] OR Caribbean Region[Mesh:noexp] OR West Indies[Mesh:noexp] OR South America[Mesh:noexp] OR Latin America[Mesh:noexp] OR Central America[Mesh:noexp] OR "Atlantic Islands"[Mesh:noexp] OR "Commonwealth of Independent States"[Mesh:noexp] OR "Pacific Islands"[Mesh:noexp] OR "Indian Ocean Islands"[Mesh:noexp] OR "Europe, Eastern"[Mesh:noexp] OR Afghanistan[Mesh] OR Albania[Mesh] OR Algeria[Mesh] OR American Samoa[Mesh] OR Angola[Mesh] OR "Antigua and Barbuda"[Mesh] OR Argentina[Mesh] OR Armenia[Mesh] OR Azerbaijan[Mesh] OR Bahrain[Mesh] OR "Baltic States"[Mesh] OR Bangladesh[Mesh] OR Barbados[Mesh] OR Benin[Mesh] OR "Republic of Belarus"[Mesh] OR Belize[Mesh] OR Bhutan[Mesh] OR Bolivia[Mesh] OR Bosnia-Herzegovina[Mesh] OR Botswana[Mesh] OR Brazil[Mesh] OR Bulgaria[Mesh] OR Burkina Faso[Mesh] OR Burundi[Mesh] OR Cambodia[Mesh] OR Cameroon[Mesh] OR Cape Verde[Mesh] OR Central African Republic[Mesh] OR Chad[Mesh] OR Chile[Mesh] OR China[Mesh] OR Colombia[Mesh] OR Comoros[Mesh] OR Congo[Mesh] OR Costa Rica[Mesh] OR Cote d'Ivoire[Mesh] OR Croatia[Mesh] OR Cuba[Mesh] OR Cyprus[Mesh] OR Czechoslovakia[Mesh] OR Czech Republic[Mesh] OR Slovakia[Mesh] OR Djibouti[Mesh] OR "Democratic Republic of the Congo"[Mesh] OR "Democratic People's Republic of Korea"[Mesh] OR Dominica[Mesh] OR Dominican Republic[Mesh] OR East Timor[Mesh] OR Ecuador[Mesh] OR Egypt[Mesh] OR El Salvador[Mesh] OR Eritrea[Mesh] OR Estonia[Mesh] OR Ethiopia[Mesh] OR "Equatorial Guinea"[Mesh] OR Fiji[Mesh] OR "French Guiana"[Mesh] OR Gabon[Mesh] OR Gambia[Mesh] OR "Georgia (Republic)"[Mesh] OR Ghana[Mesh] OR Greece[Mesh] OR Grenada[Mesh] OR Guatemala[Mesh] OR Guinea[Mesh] OR Guinea-Bissau[Mesh] OR Guam[Mesh] OR Guyana[Mesh] OR Haiti[Mesh] OR Honduras[Mesh] OR Hungary[Mesh] OR "Independent State of Samoa"[Mesh] OR India[Mesh] OR Indonesia[Mesh] OR Iran[Mesh] OR Iraq[Mesh] OR Jamaica[Mesh] OR Jordan[Mesh] OR Kazakhstan[Mesh] OR Kenya[Mesh] OR Korea[Mesh] OR Kyrgyzstan[Mesh] OR Laos[Mesh] OR Latvia[Mesh] OR Lebanon[Mesh] OR Lesotho[Mesh] OR Liberia[Mesh] OR Libya[Mesh] OR Lithuania[Mesh] OR "Macedonia (Republic)"[Mesh] OR Madagascar[Mesh] OR Malawi[Mesh] OR Malaysia[Mesh] OR Mali[Mesh] OR Malta[Mesh] OR Mauritania[Mesh] OR Mauritius[Mesh] OR "Melanesia"[Mesh] OR Mexico[Mesh] OR Micronesia[Mesh] OR Middle East[Mesh:noexp] OR Moldova[Mesh] OR Mongolia[Mesh] OR Montenegro[Mesh] OR Morocco[Mesh] OR Mozambique[Mesh] OR Myanmar[Mesh] OR Namibia[Mesh] OR Nepal[Mesh] OR Netherlands Antilles[Mesh] OR New Caledonia[Mesh] OR Nicaragua[Mesh] OR Niger[Mesh] OR Nigeria[Mesh] OR Oman[Mesh] OR Pakistan[Mesh] OR Palau[Mesh] OR Panama[Mesh] OR Papua New Guinea[Mesh] OR Paraguay[Mesh] OR Peru[Mesh] OR Philippines[Mesh] OR Poland[Mesh] OR Portugal[Mesh] OR Puerto Rico[Mesh] OR "Republic of Korea"[Mesh] OR Romania[Mesh] OR Russia[Mesh] OR "Russia (Pre-1917)"[Mesh] OR Rwanda[Mesh] OR "Saint Kitts and Nevis"[Mesh] OR Saint Lucia[Mesh] OR "Saint Vincent and the Grenadines"[Mesh] OR Samoa[Mesh] OR Saudi Arabia[Mesh] OR Senegal[Mesh] OR Serbia[Mesh] OR Montenegro[Mesh] OR Seychelles[Mesh] OR Sierra Leone[Mesh] OR Slovenia[Mesh] OR Sri Lanka[Mesh] OR Somalia[Mesh] OR South Africa[Mesh] OR Sudan[Mesh] OR Suriname[Mesh] OR Swaziland[Mesh] OR Syria[Mesh] OR Tajikistan[Mesh] OR Tanzania[Mesh] OR Thailand[Mesh] OR Togo[Mesh] OR Tonga[Mesh] OR "Trinidad and Tobago"[Mesh] OR Tunisia[Mesh] OR Turkey[Mesh] OR Turkmenistan[Mesh] OR Uganda[Mesh] OR Ukraine[Mesh] OR Uruguay[Mesh] OR USSR[Mesh] OR Uzbekistan[Mesh] OR Vanuatu[Mesh] OR Venezuela[Mesh] OR Vietnam[Mesh] OR Yemen[Mesh] OR Yugoslavia[Mesh] OR Zambia[Mesh] OR Zimbabwe[Mesh]

**Supplemental Material 2:** PubMed Search Strategy implemented for extracting records dated from 2000 - 2020

| **Database** | **Concept** | **Key Words** | | **Results** |
| --- | --- | --- | --- | --- |
| PubMed | Integrated health care model | Line 1 | (Delivery of Health Care, Integrated[mesh] OR | 499 |
|  |  | Line 2 | Integrat*[tw] OR Integrat* Care[tw] OR Integrat* Health Care[tw] OR Integrat* Healthcare[tw] OR Integrat* Health Care System*[tw] OR Integrat* Healthcare System*[tw] OR Integrat* Care Model*[tw] OR Integrat* Delivery System*[tw] OR Integrat* Service Delivery[tw] OR Integrat* Service Delivery System*[tw] OR Integrat* Health Service*[tw] OR Integrat* Health Service* Delivery[tw] OR Integrat* Health Care Polic*[tw] OR Integrat* Healthcare Polic*[tw] OR Integrat* Health Care Organization*[tw] OR Integrat* Healthcare Organization*[tw] OR Integrat* model* of health care[tw] OR Integrat* model* of healthcare[tw] OR Health System* Integrat*[tw] OR Integrat* of Health Care System*[tw] OR Integrat* of Healthcare System*[tw] OR Integrat* of Health System*[tw] OR Integrat* of Health System*[tw] OR Service* integrat*[tw] OR System* Integrat*[tw] OR |  |
|  |  | Line 3 | Continuity of Patient Care*[mesh] OR |  |
|  |  | Line 4 | Healthcare Continuum[tw] OR Health Care Continuum[tw] OR Care Continuum[tw] OR Continuum of Care[tw] OR Continuum of Healthcare[tw] OR Continuum of Health Care[tw] OR |  |
|  |  | Line 5 | Case Management*[mesh] OR |  |
|  |  | Line 6 | Care, Patient-Centered[mesh] OR Patient Centered Care[mesh] OR Patient-Centered Care*[mesh] OR Patient-Focused Care[mesh] OR Care, Patient-Focused[mesh] OR Patient Focused Care[mesh] OR |  |
|  |  | Line 7 | Coordinat*[tw] OR Coordinat* Care[tw] OR Coordinat* Health Care[tw] OR Coordinat* Healthcare[tw] OR |  |
|  |  | Line 8 | Seamless Care[tw] OR Comprehensive Health Care[tw] OR Comprehensive Healthcare[tw] OR |  |
|  |  | Line 9 | Collaborat*[tw] OR Collaboration between[tw] OR |  |
|  |  | Line 10 | Interface*[tw] OR |  |
|  |  | Line 11 | Case Manage*[tw] OR Case-management[tw] OR Case Management[tw] OR |  |
|  |  | Line 12 | Patient-Centered Care*[tw] OR Patient Centered Care*[tw] OR Patient-Focused Care*[tw] OR Patient Focused Care*[tw] OR People-centred Care*[tw] OR People Centred Care*[tw] OR People-centred health system*[tw] OR People Centred health system*[tw] OR People-centered Care*[tw] OR People Centered Care*[tw] OR People-centered health system*[tw] OR People Centered health system*[tw]) AND |  |
|  | Primary and secondary care system | Line 13 | (((Primary Care[mesh] OR Care, Primary[mesh] OR Care, Primary Health[mesh] OR Health Care, Primary[mesh] OR Primary Healthcare[mesh] OR Healthcare, Primary[mesh]) AND (Care, Secondary[mesh] OR Secondary Cares[mesh] OR Secondary Care Center[mesh] OR Secondary Referral Hospital[mesh] OR Facility, Secondary Care[mesh] OR Secondary Care Facilities[mesh] OR Secondary Referral Centers[mesh])) OR |  |
|  |  | Line 14 | ((Primary Care*[tw] OR Primary Health Care*[tw] OR Primary healthcare*[tw]) AND (Secondary Care*[tw] OR Secondary Health Care*[tw] OR Secondary healthcare*[tw]))OR |  |
|  |  | Line 15 | ((Primary Healthcare Center*[tw] OR Primary Health Care Center*[tw] Primary Care Center*[tw] OR Primary Hospital*[tw]) AND (Secondary Referral Center*[tw] OR Secondary Referral Hospital*[tw] OR Secondary Hospital*[tw])) OR |  |
|  |  | Line 15 | (Primary and Secondary[tw] OR Secondary and Primary[tw] OR Primary to Secondary [tw] OR Primary-Secondary[tw])) AND |  |
|  | Low-and middle-income countries (LMICs)^a^ | Line 17 | (Script of LMICs is presented in Supplemental Material 1) AND |  |
| Note: a = Countries Gross domestic product (GDP) less than $3,995 https://datahelpdesk.worldbank.org/knowledgebase/articles/906519-world-bank-country-and-lending-groups | | | | |

**Supplemental Material 3:** Definition of Primary-Secondary Integrated Care system

**Definition of Integrated Primary Secondary Care Services (Alma Ata):**

We did not find a comprehensive definition of an integrated primary-secondary health care model during our initial literature review. To develop the definition, we have adopted WHO’s definition of integrated health service (World Health Organization: Regional Office for Europe, 2016) and obtained additional insights from studies that explored integrated primary-secondary health care (Mitchell et al., 2015; Smith et al., 2017; Winpenny et al., 2017). The definition is as follows:

*The approach towards vertical integration of primary and secondary healthcare systems, which requires a set of coordinated strategies that involve streamlining the organizational arrangements, functional processes, service delivery apparatuses, clinical operations, and community-health facility interfaces – either by implementing independently or in any specific combinations – for incorporating secondary care functions within the primary care settings or vice versa, enabling upstream and/or downstream restructuring by augmenting health systems resources – within one setting or across health facilities – to provide evidence-based, people-centered and high-quality healthcare service and, simultaneously, to improve the performance of health systems.*

We also acknowledge that by conducting this review, we expect to gain additional insight on primary-secondary care integration, and we will actively improve the definition of the integration model. However, we do not expect this process to reduce the robustness of our analytical process and reduce the quality of the synthesis of evidence.

For the reference of the readers, we have provided our definition of primary care, secondary care, and Integrated Health Care Framework below:

| **Definition of Primary Care Services (Alma Ata):**  Primary health care is essential health care based on practical, scientifically sound, and socially acceptable methods and technology made universally accessible to individuals and families in the community through their full participation and at a cost that the community and country can afford to maintain at every stage of their development in the spirit of self-reliance and self-determination. It forms an integral part both of the country’s health system, of which it is the central function and main focus, and of the overall social and economic development of the community (“Declaration of ALMA-ATA,” 2015).  It is the first level of contact of individuals, the family, and community with the national health system bringing health care as close as possible to where people live and work and constitutes the first elements of a continuing health care process. |
| --- |
| **Definition of Secondary Care:**  Secondary Health Care is the specialist treatment and support provided by doctors and other health professionals for patients who have been referred to them for specific expert care, most often provided in hospitals (International Medical Corps, 2020). |
| **WHO Integrated Health Care Framework:**  Integrated health services delivery is defined as an approach to strengthen people-centred health systems through the promotion of the comprehensive delivery of quality services across the life-course, designed according to the multidimensional needs of the population and the individual and delivered by a coordinated multidisciplinary team of providers working across settings and levels of care. It should be effectively managed to ensure optimal outcomes and the appropriate use of resources based on the best available evidence, with feedback loops to continuously improve performance and to tackle upstream causes of ill health and to promote well-being through intersectoral and multisectoral actions (World Health Organization: Regional Office for Europe, 2016). |

**References:**

Declaration of ALMA-ATA. (2015). American Journal of Public Health, 105(6), 1094–1095. https://doi.org/10.2105/AJPH.2015.10561094

International Medical Corps. (2020). Secondary Health Care. International Medical Corps. https://www.internationalmedicalcorps.org.uk/what-we-do/all-emergencies/secondary-health-care

Mitchell, G. K., Burridge, L., Zhang, J., Donald, M., Scott, I. A., Dart, J., & Jackson, C. L. (2015). Systematic review of integrated models of health care delivered at the primary–secondary interface: How effective is it and what determines effectiveness? Australian Journal of Primary Health, 21(4), 391. https://doi.org/10.1071/PY14172

Smith, S. M., Cousins, G., Clyne, B., Allwright, S., & O’Dowd, T. (2017). Shared care across the interface between primary and specialty care in management of long term conditions. The Cochrane Database of Systematic Reviews, 2017(2). https://doi.org/10.1002/14651858.CD004910.pub3

Winpenny, E. M., Miani, C., Pitchforth, E., King, S., & Roland, M. (2017). Improving the effectiveness and efficiency of outpatient services: A scoping review of interventions at the primary–secondary care interface. Journal of Health Services Research & Policy, 22(1), 53–64. https://doi.org/10.1177/1355819616648982

World Health Organization: Regional Office for Europe. (2016). Strengthening people-centred health systems in the WHO European Region: Framework for action on integrated health services delivery. World Health Organization. http://www.euro.who.int/__data/assets/pdf_file/0004/315787/66wd15e_FFA_IHSD_160535.pdf?ua=1

**Supplemental Material 4: Description of the data extraction form**

| **Extracted data** | **Description of the data needs to be extracted** |
| --- | --- |
| Article Number | Assigned number of the article |
| Database ID | ID in the repository or database |
| Source | Name of the repository or database |
| Title | Title of the articles |
| Authors | Author of the article/report/document |
| Year | Year of publication |
| Country name | Name of the country/countries where the study/studies were/were conducted or the integration mechanisms were described |
| Country Type | - Low-income economies: GDP $1,025 or less (n = 31) - Lower-middle income economies: GDP $1,026 to $3,995 (n = 47) |
| WHO region | - African Region - Region of the Americas - South-East Asia Region - European Region - Eastern Mediterranean Region, and - Western Pacific Region |
| Study populations | If applicable |
| Study location | Urban or Rural |
| Study design and methodology | - Observational studies (qualitative, quantitative, mixed-method, case studies) - Intervention or experimental studies - Methodological report - Systemic review or scoping review - Commentary, editorial or personal narrative or review - Government document or programmatic document - Personal communication |
| Definition of integrated care | - How integrated primary-secondary health care model is defined |
| Cause of integration | What was the cause of integration |
| Typologies of integration | - Organizational: Integration of organizations are brought together formally by mergers or through ‘collectives’ the virtually through coordinated provider networks or via contacts between separate organizations brokered by the purchaser - Functional: Integration of non-clinical support and back-office functions, such as electronic patient records - Service: Integration of different clinical services at an organizational level, such as through teams of multidisciplinary professionals - Clinical: Integration of care delivered by professional and providers to patients into a single or coherent process within and/or across professions, such as through the use of shared guidelines and protocols - Mixed: Combination of two or more type above: Specify |
| Type of service integration  (if applicable) | - None - Specific Service name: Specify |
| Health systems building blocks integrated  (if applicable) | - Service Delivery - Human resource - Medicine and Technologies - Financing - Health Information - Governance - People or Communities - Specific Combinations from above: Specify |
| Mechanism of integration  (if applicable) | - Normative: based on shared values for coordination and collaboration - Systematic: coherence of rules and policies at various levels of the Organisation |
| Structure of integration  (if applicable) | - Horizontal: integration occurring across operating units and/or organizations that are at the same stage in the process of delivering services come together - Vertical: brings together organizations at different levels of the hierarchical structure under one management umbrella |
| Intensity of integration  (if applicable) | - Full integration: Process of integrating different component of the health system using a new organizational model - Partial integration: Process of creating non-binding linkages or ties support integration between health system to improve coordination between them |
| Organizational and operational components | - What was the program design, policy options used, level/s of health systems integrated, and implementation strategy |
| Success | - Effect on the health system - Effect on population health |
| Facilitators |  |
| Bottlenecks |  |
| Unintended consequences |  |
